# Rapid Identification for the Pterocarpus Bracelet by Three-Step Infrared Spectrum Method

**DOI:** 10.3390/molecules27154793

**Published:** 2022-07-27

**Authors:** Zhi Jin, Weili Cui, Fangda Zhang, Fang Wang, Shichao Cheng, Yuejin Fu, Anmin Huang

**Affiliations:** 1 Research Institute of Wood Industry, Chinese Academy of Forestry, Beijing 100091, China; zj2015@caf.ac.cn (Z.J.); zfdyale@126.com (F.Z.); 2 Fujian Inspection and Research Institute for Product Quality, Fuzhou 350001, China; z.echo87@hotmail.com; 3 Beijing Shensen Planning and Design Co., Ltd., Beijing 100123, China; 13020001826@163.com; 4 Beijing Xuekuibang Technology Co., Ltd., Beijing 100000, China; s15311918932@163.com

**Keywords:** wood identification, Pterocarpus, FTIR, 2DIR

## Abstract

In order to explore a rapid identification method for the anti-counterfeit of commercial high value collections, a three-step infrared spectrum method was used for the pterocarpus collection identification to confirm whether a commercial pterocarpus bracelet (PB) was made from the precious species of *Pterocarpus santalinus (P. santalinus)*. In the first step, undertaken by Fourier transform infrared spectroscopy (FTIR) spectrum, the absorption peaks intensity of PB was slightly higher than that of *P. santalinus* only at 1594 cm^−1^, 1205 cm^−1^, 1155 cm^−1^ and 836 cm^−1^. In the next step of second derivative IR spectra (SDIR), the FTIR features of the tested samples were further amplified, and the peaks at 1600 cm^−1^, 1171 cm^−1^ and 1152 cm^−1^ become clearly defined in PB. Finally, by means of two-dimensional correlation infrared (2DIR) spectrum, it revealed that the response of holocellulose to thermal perturbation was stronger in *P. santalinus* than that in PB mainly at 977 cm^−1^, 1008 cm^−1^, 1100 cm^−1^, 1057 cm^−1^, 1190 cm^−1^ and 1214 cm^−1^, while the aromatic functional groups of PB were much more sensitive to the thermal perturbation than those of *P. santalinus* mainly at 1456 cm^−1^, 1467 cm^−1^, 1518 cm^−1^, 1558 cm^−1^, 1576 cm^−1^ and 1605 cm^−1^. In addition, fluorescence microscopy was used to verify the effectiveness of the above method for wood identification and the results showed good consistency. This study demonstrated that the three-step IR method could provide a rapid and effective way for the anti-counterfeit of pterocarpus collections.

## 1. Introduction

*Pterocarpus santalinus* (*P. santalinus*), commonly known as “red sandalwood”, mainly originates from India, Thailand, Malaysia and Vietnam. It is a kind of rare and precious wood resource, which has been listed into the Appendix II of “Convention on International Trade in Endangered Species of Wild Fauna and Flora (CITES)” and the “Red List of International Union for Conservation of Nature (IUCN)” in order to avoid the threat of illegal logging and excessive deforestation caused by high market demand [1,2,3,4]. Considering its high commercial value and fine quality such as good mechanical performance and aesthetic appearance, *P. santalinus* has been used to manufacture valuable collections like luxury furniture and top crafts, etc [5,6]. Driven by extremely high profit, some illegal merchants use other common and low cost species belonging to the genus *Pterocarpu*, such as *P. macrocarpus*, *P. erinaceus* and *P. tinctorius*, to fake the real *P. santalinus,* as the macro and micro characteristics of these species are too similar to be identified immediately and precisely [7]. Thus, it is necessary to develop an efficient and reliable way to distinguish these similar species for commercial trade regulation.

The existing methods of wood recognition are mainly based on anatomy, genetic biology and chemotaxonomical analysis. Traditional anatomy identification is well-established and most frequently used, which distinguishes wood samples by comparing their macroscopic and microscopic anatomical features [8,9,10], but it cannot accurately discriminate wood samples at the species scale [11,12]. Although the genetic method of DNA barcoding has been demonstrated to be effective in wood recognition even at the species scale, its wide application is limited by some technological challenges concerning DNA extraction, barcode selection and reference database [13,14,15], whereas the chemotaxonomical analysis exhibits more flexibility and potential for efficient identification, which is mainly based on various qualitative and quantitative fingerprint information of wood samples or their extractives obtained by chemical characterization techniques involving mass spectrometry [3], fluorescence [16,17], nuclear magnetic resonance spectroscopy [18], Fourier transform infrared spectroscopy (FTIR) [19,20] or a specific combination of some of the above [21,22,23]. However, due to tedious sample preparation such as purification and separation, some of these techniques are time consuming [24]. In addition, the criteria for discrimination by chemotaxonomical approaches have still not been authoritatively created [25,26]. It has been demonstrated that the operability and reliability of these techniques need to be further enhanced for rapid and accurate wood specie identification.

It is well known that the FTIR spectrum can determine the functional groups of a complex intermixture system in a holistic manner, while second derivative IR spectra (SDIR) is obtained by differential processing of the original FTIR spectrum, which can make the original peak more acute and allow it to be identified more clearly [27,28,29]. In comparison with FTIR and SDIR, two-dimensional correlation infrared (2DIR) can present higher resolution and reveal more information of interactions among intramolecular functional groups and intermolecular by means of a cross correlation analysis in a series of dynamic infrared spectra obtained through applying external perturbation to the test sample, which has been widely used in the domains of traditional Chinese medicine identification, polymer material analysis, etc. [30,31,32]. With the combination of SDIR and 2DIR spectroscopy into FTIR spectrum, the distinctions in the chemical fingerprint characteristics of different samples can be effectively amplified. Our team has innovatively established a “three-step identification” concept for wood identification by combining the above three infrared spectroscopy methods since 2008 [33]. The three-step identification only requires a very small amount of specimen which can be detected directly without tedious pre-processing procedures, so it is a rapid and non-destructive method suitable for wide application. Its feasibility and effectiveness for wood identification has also been confirmed constantly. Huang et al. [33] first proved that the 2DIR spectroscopy could be a new method to discriminate *Dalbergia dorifera*, *P. santalinus* and *P. soyauxii.* Subsequently, Zhang et al. [34,35] and Wang et al. [36] successfully employed a three-step IR identification method to distinguish *P. santalinus*, *Dalbergia louvelii*, *Dalbergia cochinchinensis*, *Dalbergia retusa*, *Dalbergia bariensis*, *Dalbergia oliveri* and their extractives with minimum samples in a direct, rapid and holistic manner. Liu et al. [37] showed that it was possible to identify *P. santalinus*, *P. tinctorius* and *Dalbergia louvelii* with wood wax oil by a three-step IR method to avoid consumer confusion. On the basis of the above research, the three-step IR approach for the commercial pterocarpus bracelet (PB) identification will be studied in this paper to explore the applicability of the three-step IR rapid identification for the anti-counterfeit of commercial high value collections. Additionally, fluorescence microscopy was used to verify the effectiveness of the above method.

## 2. Results and Discussion

### 2.1. FTIR Analysis

**Figure 1:**
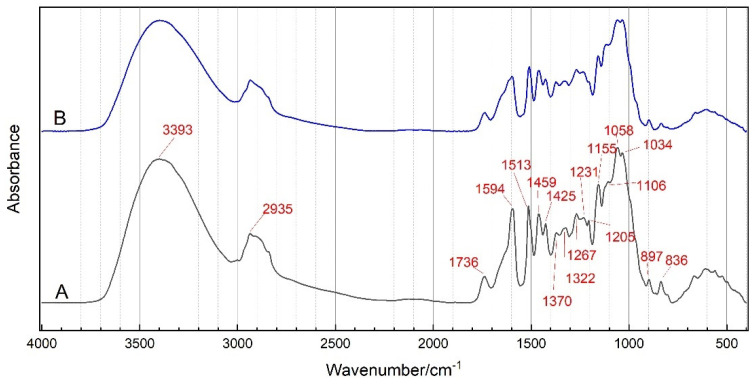
Fourier transform infrared spectroscopy (FTIR) spectra of pterocarpus bracelet (PB) (**A**) and *P. santalinus* (**B**).

The FTIR spectra of PB and *P. santalinus* are compared in Figure 1. As can be seen, the FTIR spectra of PB and *P. santalinus* have a high similarity in peak position and area. The main characteristic FTIR bands of PB and *P. santalinus* are shown in Table 1. The absorption peaks intensity of PB is slightly higher than that of *P. santalinus* only at 1594 cm^−1^, 1205 cm^−1^, 1155 cm^−1^ and 836 cm^−1^, corresponding to the stretching vibration of carbon atoms in the aromatic framework, C-O-C stretching vibration in extractives, C-O-C antisymmetric stretching vibration in cellulose and hemicellulose, and the C-H out-of-plane deformation of the aromatic ring in extractives and lignin, respectively.

### 2.2. SDIR Analysis

**Figure 2:**
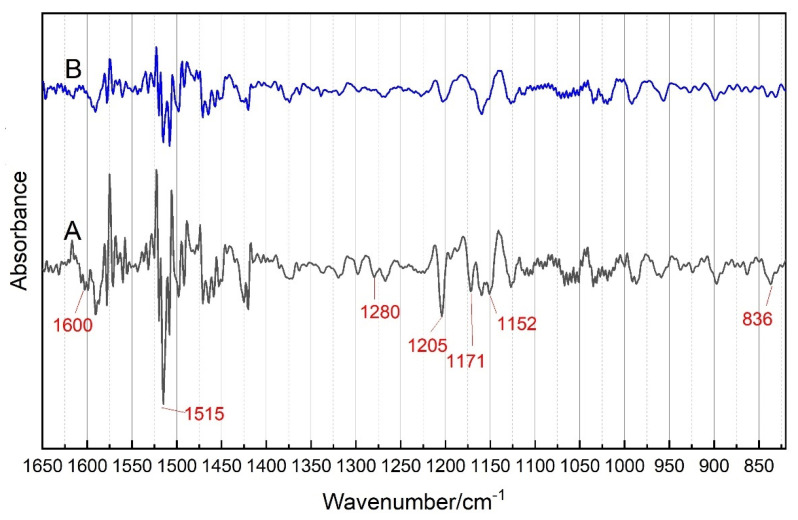
Second derivative IR spectra (SDIR) spectra of pterocarpus bracelet (PB) (**A**) and *P. santalinus* (**B**).

Figure 2 presents the SDIR spectra of PB and *P. santalinus*. According to the slope of the FTIR absorption peaks, the FTIR features of the tested samples are amplified by SDIR, which improves the spectrum apparent resolution and makes the differences in the one-dimensional FTIR spectra more recognizable. Compared with FTIR results, the differences in the absorption peak intensity at 1205 cm^−1^ and 836 cm^−1^ between PB and *P. Santalinus* are more obvious in the SDIR spectra. Notably, the shoulder peak around 1594 cm^−1^ and some overlapped absorption peaks around 1155 cm^−1^ in the FTIR spectra are also separated by using SDIR. As shown in the SDIR spectrum of PB, the peaks at 1600 cm^−1^, 1171 cm^−1^ and 1152 cm^−1^ become clearly defined. These newly presented peak positions provide more evidence for distinguishing these two samples in addition to the information obtained from the FTIR spectrum.

### 2.3. 2DIR Analysis

**Figure 3:**
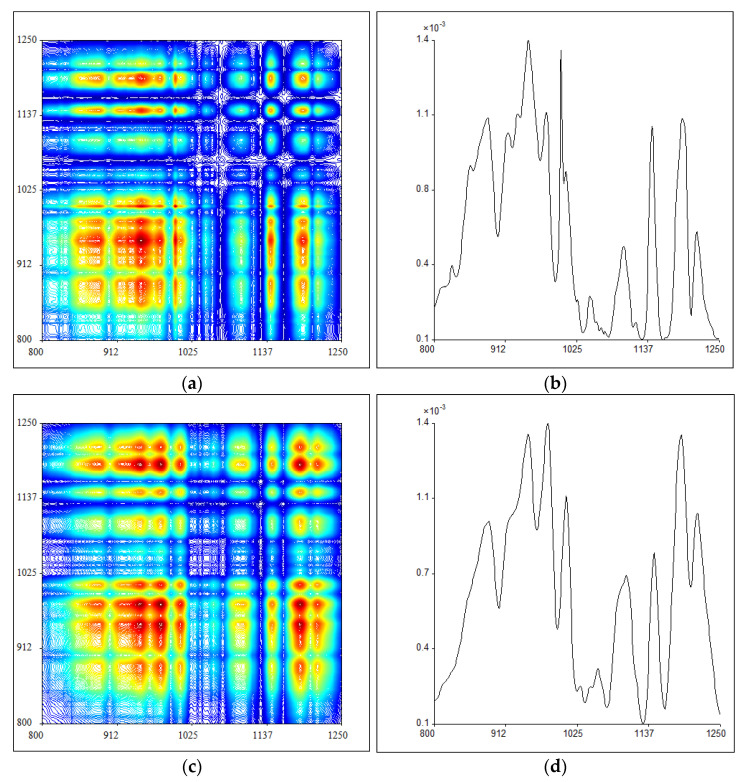
The synchronous two-dimensional correlation infrared (2DIR) spectra of pterocarpus bracelet (PB) (**a**) and *P. santalinus* (**c**) at 800–1250 cm^−1^ and the corresponding automatic peaks distribution of pterocarpus bracelet (PB) (**b**) and *P. santalinus* (**d**).

In the third step, the infrared spectral resolution is further enhanced by applying thermal perturbation to the samples and using two-dimensional correlation analysis to form 2DIR spectra. Since different chemical functional groups make different responses to temperature variations, more information can be captured for identification with external thermal perturbation. The synchronous 2DIR spectra of PB and *P. santalinus* at 800–1250 cm^−1^ are shown in Figure 3a,c. The distribution for a group of automatic peaks on the main diagonal of the above spectra is shown on the right (Figure 3b,d). These automatic peaks show the self-correlativity and susceptibility of some normal vibration of functional groups as the external temperature is increased, of which the intensity is proportional to the response of functional groups to thermal perturbation. The results show that the 2DIR spectra of PB and *P. santalinus* in this band are similar, and there is no great difference in the number of automatic peaks. The automatic peak intensity at 800–1250 cm^−1^ for PB and *P. santalinus* is compared in Table 2. It can be seen that *P. santalinus* shows a weak peak at 1057 cm^−1^ and several peaks at 977 cm^−1^, 1008 cm^−1^, 1100 cm^−1^, 1190 cm^−1^ and 1214 cm^−^^1^ with stronger intensity than those of PB. The region around 1000–1200 cm^−1^ mainly covers the C–O stretching vibration from carbohydrate [40,41], thus it reveals that the response of holocellulose to thermal perturbation is stronger in *P. santalinus* than that in PB.

**Figure 4:**
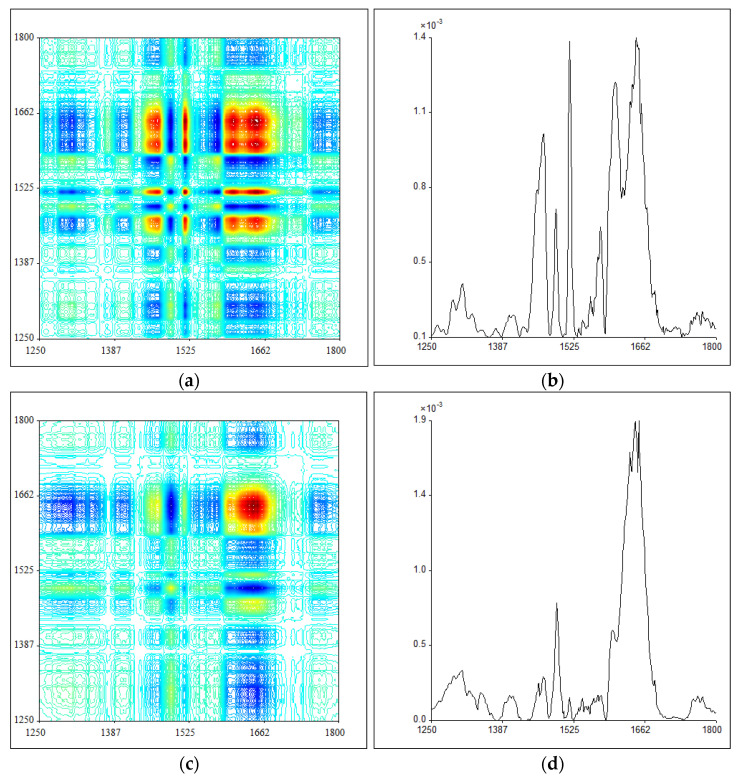
The synchronous two-dimensional correlation infrared (2DIR) spectra of pterocarpus bracelet (PB) (**a**) and *P. santalinus* (**c**) at 1250–1800 cm^−1^ and the corresponding automatic peaks distribution of pterocarpus bracelet (PB) (**b**) and *P. santalinus* (**d**).

Another region of 2DIR (1250–1800 cm^−1^) and the corresponding automatic peak distribution for PB and *P. santalinus* is presented in Figure 4, and their automatic peak positions and intensity are summarized in Table 3. By comparison, the difference between the 2DIR results of PB and *P. santalinus* in this range is much more evident than that at 800–1250 cm^−1^. It can be seen that there is a strong peak at 1518 cm^−1^, a middle strong peak at 1576 cm^−1^, and a weak peak at 1558 cm^−1^ for PB, which are almost negligible for *P. santalinus*. Moreover, the PB shows two strong peaks at 1467 cm^−1^ and 1605 cm^−1^, and two middle strong peaks at 1456 cm^−1^ and 1576 cm^−1^, while in the case of *P. santalinus* these peaks are rather weak. All these characteristic autopeaks are mainly attributed to the aromatic ring skeleton vibration, suggesting that the aromatic functional groups of PB are much more sensitive to the thermal perturbation, which can be used to effectively identify these two samples.

### 2.4. Fluorescence Microscopy

**Figure 5:**
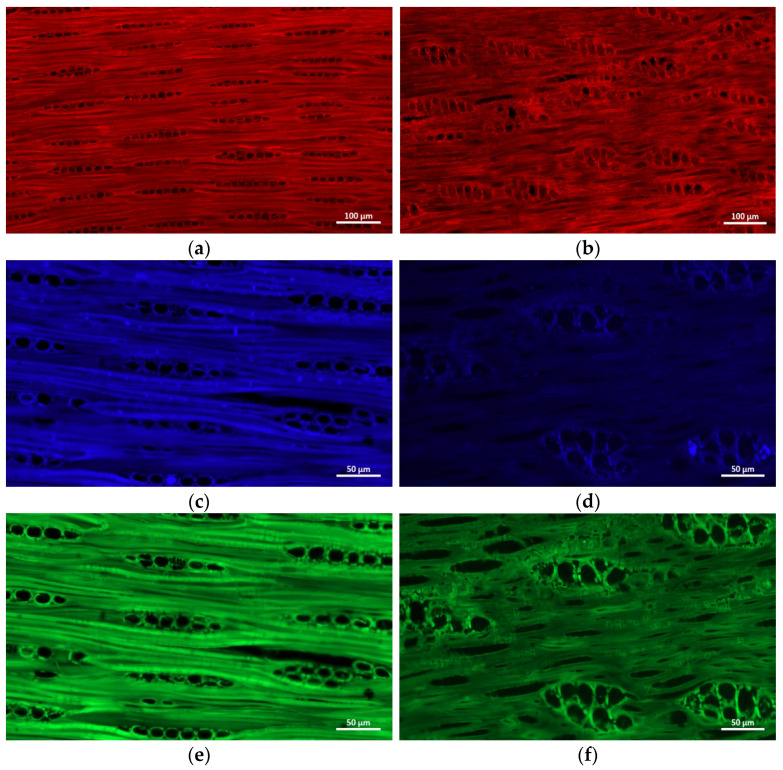
The auto-fluorescence images of the longitudinal sections of pterocarpus bracelet (PB) (**a**,**c**,**e**) and *P. Santalinus* (**b**,**d**,**f**) at different excitation wavelengths (**a**,**b**: 488 nm; **c**,**d**: 405 nm; **e**,**f**: 500 nm).

Fluorescence microscopy is used to verify the effectiveness of the above three-step IR method. The auto-fluorescence images of the longitudinal sections of PB and *P. Santalinus* at different excitation wavelengths are shown in Figure 5. It can be seen that the fluorescence characteristics of these two samples is quite different. At excitation wavelength of 488 nm, the cell wall fluorescence intensity of these two samples is similar (Figure 5a,b), while at excitation wavelengths of 405 nm and 500 nm, the fluorescence intensity of the PB cell wall is obviously higher than that of *P. Santalinus* (Figure 5c–f), which is consistent with the FTIR results showing that PB exhibits a stronger aromatic framework stretching vibration. Interestingly, there exists numerous cell inclusions with higher fluorescence intensity within the ray parenchyma lumen for *P. Santalinus* than that for PB at the excitation wavelength of 488 nm. This suggests a stronger fluorescence reaction for the *P. Santalinus* extracts, which has been used as an important feature for distinction among pterocarpus wood species [42].

## 3. Materials and Methods

### 3.1. Materials and Samples Preparation

The pterocarpus bracelet was purchased online. Samples were scraped directly from the core of the bracelet. The *P. santalinus* specimen was provided by the Research Institute of Wood Industry, Chinese Academy of Forestry, China. These samples were air- dried and grounded into powder. Then, each wood sample (2 mg) was mixed with KBr (100 mg) and grounded again in the grinder. Finally, the mixture was dried and pressed into a thin disk for spectra analysis.

### 3.2. FTIR Analysis

FTIR spectra were recorded by 16 scans in 4000–400 cm^−1^ under a resolution of 4 cm^−1^ using a spectrometer (Spectrum GX FTIR, PerkinElmer Inc., Waltham, MA, USA) equipped with a DTGS detector.

### 3.3. SDIR Analysis

The second derivative IR spectra were obtained by using the derivative function of Spectrum to Window software (Perkin–Elmer Corporation, Waltham, MA, USA).

### 3.4. 2DIR Analysis

2DIR spectra were collected at 50–120 °C with an interval of 10 °C using 2DIR software (Tsinghua University, Beijing, China). The temperature perturbation was performed by a portable programmable temperature controller (Model 50-886, Love Control, Buffalo, NY, USA).

### 3.5. Fluorescence Microscopy

Longitudinal sections (15-μm thick) were cut from the each wood block using a sliding microtome (Leica RM2010R, Leica Microsystems GmbH, Wetzlar, Germany). The autofluorescence signals of the sections were observed using an Axio Imager M2 microscope (Zeiss, Jena, Germany) under 405 nm, 488 nm and 500 nm excitation light. The emission wavelength at 422 nm (blue), 630 nm (green), and 525 nm (red) was used.

## 4. Conclusions

A three-step infrared spectrum method was used to confirm whether a commercial pterocarpus bracelet (PB) is made from the precious species of *P. santalinus*. In the first step by FTIR spectrum, the absorption peaks intensity of PB is slightly higher than that of *P. santalinus* only at 1594 cm^−1^, 1205 cm^−1^, 1155 cm^−1^ and 836 cm^−1^. In the second step, the FTIR features of the tested samples are further amplified by SDIR, and the peaks at 1600 cm^−1^, 1171 cm^−1^ and 1152 cm^−1^ become clearly defined in PB so as to provide more evidence for distinguishing these two samples. Finally, 2DIR spectra reveals that the response of holocellulose to thermal perturbation is stronger in *P. santalinus* than that in PB, mainly at 977 cm^−1^, 1008 cm^−1^, 1100 cm^−1^, 1057 cm^−1^, 1190 cm^−1^ and 1214 cm^−1^, while the aromatic functional groups of PB are much more sensitive to the thermal perturbation than those of *P. santalinus,* mainly at 1456 cm^−1^, 1467 cm^−1^, 1518 cm^−1^, 1558 cm^−1^, 1576 cm^−1^ and 1605 cm^−1^. All of the above differences between PB and *P. santalinus* successively demonstrated by the FTIR, SDIR and 2DIR spectrum provide a rapid and effective way for the anti-counterfeit of pterocarpus collections. In addition, fluorescence microscopy results further confirm the effectiveness of the above three-step infrared spectrum method for wood identification.

## Figures and Tables

**Table 1:** The main characteristic Fourier transform infrared spectroscopy (FTIR) bands of pterocarpus bracelet (PB) (A) and *P. santalinus* (B) [34,35,36,38,39].

Wavenumber (cm^−1^)		Band Assignment
A	B	
3399	3399	O–H stretching vibration of carbohydrate C–OH
2935	2934	C–H asymmetric stretching in alkanes (methyl or methylene)
1736	1735	Unconjugated C=O stretching vibration of xylan
1594	1596	Stretching vibration of carbon atoms in the aromatic framework
1513	1508	Aromatic skeletal vibrations in extractives and lignin
1459	1459	C–H bending vibration; aromatic skeletal vibrations
1425	1426	Aromatic skeletal vibration in lignin and C–H deformation in-plane deforming
1370	1373	C–H deformation, CH3 symmetric deformation in holocellulose
1322	1328	C–O stretch of acetate group in hemicelluloses and interaction band involving C–OH bend
1267	1267	O–C–O and Guaiacyl ring Stretching vibration in lignin and xylan
1231	1234	O–C–O and Syringyl ring Stretching vibration in lignin and xylan
1205	1205	C–O–C stretching vibration in extractives
1155	1157	C–O–C stretching or frame vibration in holocellulose
1106	1112	C–C, C–O stretching in holocellulose
1058	1059	C–O stretching vibration in holocellulose
1034	1034	C–O stretching vibration
897	898	C1–H deformation in cellulose
836	834	C–H out-of-plane deformation of aromatic ring in extractives and lignin

**Table 2:** Autopeak intensity in the two-dimensional correlation infrared (2DIR) spectra of pterocarpus bracelet (PB) (A) and *P. santalinus* (B) at 800–1250 cm^−1^.

	828	885	916	949	977	1000	1008	1045	1057	1100	1144	1190	1214
A	+	++	++	+++	++	+++	+	+	−	+	++	++	+
B	−	++	++	+++	+++	++	++	+	+	++	+	+++	++

−, invisible; +, weak; ++, middle; +++, strong.

**Table 3:** Autopeak intensity in the two-dimensional correlation infrared (2DIR) spectra of pterocarpus bracelet (PB) (A) and *P. santalinus* (B) at 1250–1800 cm^−1^.

	1292	1311	1456	1467	1491	1518	1558	1576	1605	1645
A	+	+	++	+++	++	+++	+	++	+++	+++
B	+	+	+	+	++	−	−	−	+	+++

−, invisible; +, weak; ++, middle; +++, strong.

## Data Availability

Conform to “MDPI Research Data Policies” at https://www.mdpi.com/ethics (accessed on 26 May 2022).

## Sample Availability

The wood samples used are available from the authors.
